# Demethyltransferase FTO alpha-ketoglutarate dependent dioxygenase (FTO) regulates the proliferation, migration, invasion and tumor growth of prostate cancer by modulating the expression of melanocortin 4 receptor (MC4R)

**DOI:** 10.1080/21655979.2021.2001936

**Published:** 2022-02-19

**Authors:** Sheng Li, Lin Cao

**Affiliations:** a Department of Urology Surgery, Ningbo Medical Centre LiHuiLi Hospital, Zhejiang, Ningbo, China; b Department of Urology, Xiuzhou District People's Hospital of Jiaxing, Zhejiang, Jiaxing, China

**Keywords:** Prostate cancer, FTO, MC4R, M6A, malignant progression

## Abstract

N6-Methyladenosine (m6A) is the most abundant modifications in human messenger RNAs (mRNAs). This study aimed at investigating the function and mechanism of demethyltransferase fat mass and obesity-associated protein (FTO) in prostate cancer(PCa). The expression level of FTO in PCa was detected by quantitative reverse transcription-polymerase chain reaction (qRT-PCR) and Western blot. Besides, the impacts of FTO on the proliferation, migration and invasion of PCa cells were also detected by cell counting kit-8 (CCK-8), 5-ethynyl-2ʹ-deoxyuridine (EdU) and transwell assays. Furthermore, we also explored the potential mechanism of FTO in PCa. The results showed that FTO expression was decreased in PCa, and the low expression of FTO showed an obvious relevance to the clinical characteristics. Downregulation of FTO facilitated the proliferation, migration, invasion and tumor growth of PCa cells. Besides, MC4R displayed a remarkably high expression in PCa tissues, whose expression and m6A level were regulated by FTO. Meanwhile, the in vitro experiments revealed that highly expressed FTO partially reversed the facilitating effect of highly expressed MC4R on the malignant phenotype of PCa cells. Overall, FTO was downregulated in PCa and its expression level showed a relevance to the prognosis of PCa patients. Additionally, FTO could regulate the proliferation, migration and invasion of PCa via regulating the expression level of MC4R.

## Introduction

Prostate cancer (PCa), one of the world’s most common malignancies with high morbidity (ranking 2nd) and high mortality (ranking 6th), is frequently diagnosed in Europe and the United States, and the number of new male cases of PCa has become the largest [1]. There are approximately 1.27 million new cases every year, and approximately 360,000 people die of PCa every year [2]. The disease can mainly be treated by surgery, radiotherapy and chemotherapy, endocrine therapy, among others, but patients in the advanced-stage cannot be cured. With the development in molecular biology, targeted gene therapy has become a new treatment method for malignancies [3]. Metastasis is the main cause of morbidity and mortality of PCa [4,5]. Hence, exploring the molecular mechanism of the occurrence and development of PCa may be highly valuable for the early diagnosis and molecular targeted therapy of PCa.

N6-methyladenosine (m6A) modification refers to the methylation of the sixth nitrogen atom of the RNA molecule adenylate. Methylation modification of m6A is one of the most abundant mRNA modifications in higher eukaryotes [6]. Similar to DNA methylation, RNA methylation is catalyzed by methyltransferases, which transfer methyl groups from S-adenosylmethionine to RNA-specific methylation sites [7]. The m6A methylation modification has been found to be widespread in many species such as animals and plants, prokaryotes, and viruses. m6A is mainly distributed in the protein coding sequence (CDS) of messenger RNA (mRNA), near the splice point, and the 3ʹuntranslated region (3ʹ UTR) near the stop codon. The study of m6A modification sites has shown that m6A modification affects the process of mRNA transcription, splicing, nucleation, translation, and degradation [8]. m6A can regulate messenger RNAs (mRNAs) expression by reversible process, which is promoted by writers, inhibited by erasers, and executed by readers [9,10]. Recently, an increasing number of studies have demonstrated that writers, erasers, and readers perform vital biological functions in human malignancies. For examples, YTHDF2, as an m6A reader, usually recognizes m6A in the 3ʹ-UTR of mRNAs, which leads to mRNA degradation [11]. ALKBH5 suppresses the occurrence of PCa by reducing the methylation level of *WIF-1* RNA and mediating the Wnt signaling pathway [12]. Additionally, METTL14 can inhibit the proliferation, migration, and invasion of gastric cancer by regulating the PI3K/AKT/mTOR signaling pathway [13].

Fat mass and obesity-associated protein (FTO) gene is the first obesity susceptibility gene confirmed by whole genome scanning method. It is located on human chromosome 16q12.2, with a total length of about 430kb, containing 9 exons and 8 internal Intron, widely expressed in hypothalamus, adipose tissue, pancreatic islets and other tissues [14,15]. FTO was revealed as the first N6-methyladenosine (m6A) demethylation of eukaryotic messenger RNA (mRNA) Enzymes, and the role of FTO in adipogenesis and tumorigenesis is related to its m6A demethylase activity [16]. Many studies have demonstrated that FTO can act as a vital role in the occurrence and progression of various tumors. For examples, FTO can decrease m6A methylation of LINC00022 transcript, contributing to the suppression of LINC00022 decay via the m6A reader YTHDF2 in esophageal squamous cell carcinoma (ESCC), thereby promoting the cell proliferation and tumor growth of ESCC [17]. FTO silencing can increase the methylation level of eIF4G1, and YTHDF2 can recognize the methylation site of eIF4G1, which leads to mRNA degradation and reduced eIF4G1 protein expression, thereby promoting autophagy and reducing the occurrence of oral squamous cell carcinoma (OSCC) [18]. Studies also indicate that FTO is downregulated in PCa tissues and can act as a tumor suppressor to repress cell proliferation, migration and invasion [19,20]. However, the potential mechanism of FTO in PCa remains to be further explored.

In the current research, we suspected that FTO could act as a tumor suppressor on PCa by regulating the expression of MC4R via a m6A RNA demethylation manner. Therefore, the aim of our study was to demonstrate the interaction and biological function of FTO and MC4R in PCa, which might provide novel sights into diagnostic biomarkers and therapeutic targets for PCa.

## Materials and methods

### Online database analysis

The expression level of FTO and MC4R in PCa tissues (n = 497) and normal tissues (n = 52) were analyzed by UALCAN database (http://ualcan.path.uab.edu/analysis). The prognostic value of FTO (overall survival and disease-free survival) in glioma patients were analyzed in a downloaded profile from GEPIA dataset (http://gepiacancer-pku.cn/index.html).

### Sample collection

In this study, 50 PCa specimens and 50 benign prostatic hyperplasia (BPH) patients specimens were collected from the Ningbo Medical Center LiHuiLi Hospital and Zhejiang Veteran Hospital. None of the patients underwent preoperative chemotherapy or radiotherapy. The collected tissues were frozen at −80°C and stored in liquid nitrogen. Tumor pathological classification and staging were conducted according to the standards formulated by the Union for International Cancer Control (UICC). All patients signed the informed consent form, and this study was approved by the Ethics Committee and conformed to the Helsinki Declaration.

### Cell culture

Human normal prostate cell line (WPMY-1) and PCa cell lines (PC-3, DU145, 22Rv1, LNCap) were obtained from ATCC (ATCC Cell Resource Center, Manassas, VA, USA). All the cells were cultured in RPMI 1640 medium (Life Technologies) with 10% fetal bovine serum (FBS) (Gibco, Thermo Fisher Scientific, Inc.) and 1% double antibodies (penicillin/streptomycin). Later, these cell lines were preserved in a humidified cell incubator with 5% CO_2_ at 37°C.

### Cell transfection

Short hairpin RNAs (shRNAs) targeting FTO and MC4R constructs were supplied by RiboBio (Guangzhou, China), and the overexpression plasmids of FTO and MC4R (FTO OE and MC4R OE) were purchased from GenePharma (Shanghai,China). After the cell fusion rate reached 70–80%, lentivirus packaging plasmids were utilized for cell transfection. For overexpressing or silencing of FTO, overexpressed plasmid or shRNA target FTO or the negative control was inserted into pLKO.1 vector (Biosettia). 293 T cells (4 × 10^5^/well) were cotransfected with pLKO- FTO (or pLKO-sh- FTO) or pLKO-NC with psPAX2 and pMD2.G by Lipofectamine 2000 (Invitrogen). 48 hours later, lentiviruses were harvested. Cell transfections were performed with the Lipofectamine 2000 (Invitrogen) according to the manufacturer’s protocol [21]. The cells undergoing transfection were all stored in a humidified cell incubator with 5% CO_2_ at 37°C. The sequences of shRNAs for FTO and MC4R were listed in Table 1.

**Table 1.** Sequences of primers for transfection

Name		Sequence
FTO shRNA	Sense	5ʹCACCGCAGCTGAAATATCCTAAACTCGAAAGTTTA GGATATTTCAGCTGC −3’
	Anti-sense	5ʹAAAAGCAGCTGAAATATCCTAAACTTTCGAGTTTA GGATATTTCAGCTGC −3’
FTO NC	Sense	5ʹ- CCGGCAACAAGATGAAGAGCAC CAACTCGAGTTGGTGCTCTTCATCTTGTTGTTTTTG −3’
	Anti-sense	5ʹ- AATTCAAAAACAACAAGATGA AGAGCACCAACTCGAGTTGGTGCTCTTCATCTTGTTG −3ʹ
MC4R shRNA	Sense	5ʹ-CACCGCTGGTGAGCGTTTCAAATGGCGAACCATTTGA AACGCTCACCAGC −3’
	Anti-sense	5ʹ- AAAAGCTGGTGAGCGTTTCAAATGGTTCGCCATTTGAAA CGCTCACCAGC −3’
MC4R NC	Sense	5ʹ- CCGGCAACAAGATGAAGAGCAC CAACTCGAGTTGGTGCTCTTCATCTTGTTGTTTTTG −3’
	Anti-sense	5ʹ- AATTCAAAAACAACAAGATGA AGAGCACCAACTCGAGTTGGTGCTCTTCATCTTGTTG −3ʹ

### Quantitative reverse transcription-polymerase chain reaction (qRT-PCR)

With reference to the product specification, the total RNAs were extracted from tissues or cells with TRIzol reagent (Invitrogen, Newwork, USA) and then subjected to reverse transcription into complementary DNA (cDNA) using PrimeScript RT reagent kit (Takara, Dalian, China) in accordance with the manufacturer’s instructions [22]. Subsequently, real-time PCR analysis was carried out using SYBR Green (Takara Biotechnology Inc.). The reactions were completed under the following conditions: at 94°C for 2 min, and with 50 cycles of 94°C for 5 s, 58°C for 10 s, 72°C for 30 s, and 72°C for 10 min. The relative expression was calculated using the 2^−ΔΔCT^ method [22], and GAPDH was used as the internal reference. The experiment was repeated three times. All the primers were synthesized by Coweldgen Scientific Co., Ltd. Primer sequences are shown below:

FTO: F: 5ʹ-ACTTGGCTCCCTTATCTGACC-3ʹ

R: 5ʹ-TGTGCAGTGTGAGAAAGGCTT-3ʹ

MC4R:F:5ʹ-CTGATGGAGGGTGCTACGAG-3ʹ

R: 5ʹ- TGGGTGAATGCAGATTCTTGTT-3ʹ

GAPDH: F: 5ʹ-CTCGCTTCGGCAGCACA-3ʹ

R: 5ʹ-ACGCTTCACGAATTTGCGT-3ʹ

### Cell counting kit-8 (CCK-8) assay

According to the product specification, the CCK-8 kit (CCK-8, cat. no. C0037, Beyotime, China) was used to detect the proliferation abilities of PC-3 and DU145 cells according to manufacturer’s instructions [23]. In brief, PC-3 and DU145 cells undergoing transfection were inoculated into a 96-well plate at a density of 5 × 10^3^ cells/well, and each well was added with 10 μL of CCK-8 reagent was added to each well at the specified time points (0, 24, 48, 72, 96 h) for 2-h incubation in the dark. Finally, the absorbance at the wavelength of 450 nm was measured using an Epoch Microplate Reader (BioTek, Winooski, VT, USA)

### 5-ethynyl-2ʹ-deoxyuridine (EdU) assay

Following 24-h transfection, the proliferation abilities of PC-3 and DU145 cells were examined using EdU kit (Click-iT® EdU Imaging Kits, Invitrogen) as per the product specification [24]. Subsequent to transfection, the cells were cultured in a 96-well plate at a density of 5 × 10^3^/well, incubated for 3 h with 10 μL of EdU reagent per well, and fixed with 4% formaldehyde at room temperature for 20 min. After formaldehyde was washed away with PBS, the cells were incubated with 0.5% Triton X-100 (Sigma, San Francisco, CA, USA) at room temperature for 20 min and stained with DAPI (4ʹ,6-diamidino-2-phenylindole). In the end, the stained cells were photographed and counted by a fluorescence microscope (CKX41-F32FL, Olympus, Tokyo, Japan).

### Transwell assay

The transwell migration and invasion assays were performed using transwell chambers, as described previously [25]. Briefly, at 24 h post-transfection, the cells were digested, collected, counted, and resuspended in RPMI⁃1640 culture medium without FBS, with cell density of 2 × 10^5^/mL. Then 200 μL of the above cell suspension was inoculated into the upper Transwell chamber (Corning Incorporated, Corning, NY., USA) and RPMI⁃1640 culture medium containing 10% FBS in the lower chamber, which were placed into a cell incubator for 24-h in conventional culture. After washing twice with PBS, the cells were fixed with 4% paraformaldehyde at room temperature for 10 min, and stained with crystal violet for 20 min. Following washing off the excess crystal violet on the chamber with PBS, the cells were dried atat room temperature. Later, the cells were observed under an inverted microscope, counted and photographed. Furthermore, the migration assay required no matrix gel, but the invasion assay required matrix gel on the upper chamber, and the rest procedures of the two assays were the same.

### A nude mouse xenograft model

PCa cells carrying the transfected plasmid were subcutaneously injected into immunodeficient mice at a rate of 1 × 10^6^ cells per mouse according to a previous study [26]. Tumor formation in the two groups of mice was observed and recorded by a designated personnel every day, and the volume of the tumors was measured. The nude mice were sacrificed 21 days after tumor formation, and the tumors were removed to measure their volume and weight. The nude mice used in this experiment were SPF BALB/C nude mice aged 4–6 weeks from the Shanghai Institute of Zoology, Chinese Academy of Sciences.

### Western blot assay

The Western blot assay was conducted according to a previous study [22]. Briefly, Transfected PC-3 and DU145 cells were digested and resuspended, and then inoculated into a 6-well plate at a density of 2 × 10^6^/well. Then, the protein expression levels in the cells were detected after cell culture for 24 h. Total cell proteins were extracted with protein lysate (RIPA: PMSF = 100: 1) and measured by bicinchoninic acid (BCA) assay. Afterward, 60 μg of proteins from each group were electrophoresed and transferred onto PVDF membranes. After sealing at room temperature for 1 h, the proteins were incubated with primary antibodies at low temperature overnight and with secondary antibodies labeled with horseradish peroxide the next day. At 1 h post-incubation, the color was developed by adding a chromogenic solution and photographed for analysis. The antibodies against FTO (1/1000, ab280081) and MC4R (1/2000, ab150419) were obtained from Abcam (Cambridge, MA, USA). HRP-conjugated secondary goat anti-mouse and goat anti-rabbit were purchased from Proteintech (Proteintech, USA). The level of protein expression was detected by ECL Plus (Millipore, USA) using a Bio-Imaging System (Bio-Rad, USA).

### Dual-luciferase reporter gene assay

The firefly luciferase plasmids of MC4R-3ʹ UTR, with the wild-type m6A motifs and mutant m6A motifs (A was replaced by C), were designed by GeneChem (Shanghai, China). Brifely, the firefly luciferase plasmids of wild-type or mutant m6A motifs of MC4R 3ʹ UTR, FTO shRNA and overexpression plasmids, or GV657-flag and Ranilla plasmids were jointly transferred to PCa cells. Cells were measured by utilizing the dual-luciferase reporter assay kit (Promega, USA) [27].

### m6A immunoprecipitation (MeRIP) assay

Magna MERIP m6A Kit (Millipore, USA) was applied to carry out the MeRIp assay according to the manufacturer’s instructions [28]. Anti-m6A body (ab208577, Abcam, USA), anti-FTO body (ab252562, Abcam, USA) and anti-DDDK tag body ((ab205606, Abcam, USA) were aoolied for MeRIP detection.

### Statistic analysis

SPSS software (version 17.0) was used for data analysis. GraphPad Prism software was used for photo editing. Statistical analysis was performed using the t-test to evaluate differences in the experimental data. Statistical significance was set at p < 0.05.

## Results

In this study, we found that the m6A demethyltransferase FTO might regulate the expression of MC4R via a m6A manner in PCa by bioinformatics analysis. Hence, we intended to explore the biological function of the FTO/MC4R axis in PCa. The results indicated that FTO was downregulated in PCa, and knockdown of FTO promoted the proliferation, migration and invasion of PCa cells. While the expression level of MCR4 was increased in PCa tissues and overexpression of MCR4 could promote the malignant progression of PCa. Moreover, FTO could inhibit MC4R expression in a m6A manner. In summary, our findings revealed the function of the FTO/MC4R axis in PCa cells, which might provide a novel insight for PCa diagnosis and therapy.

### FTO was downregulated in PCa

**Figure 1.**
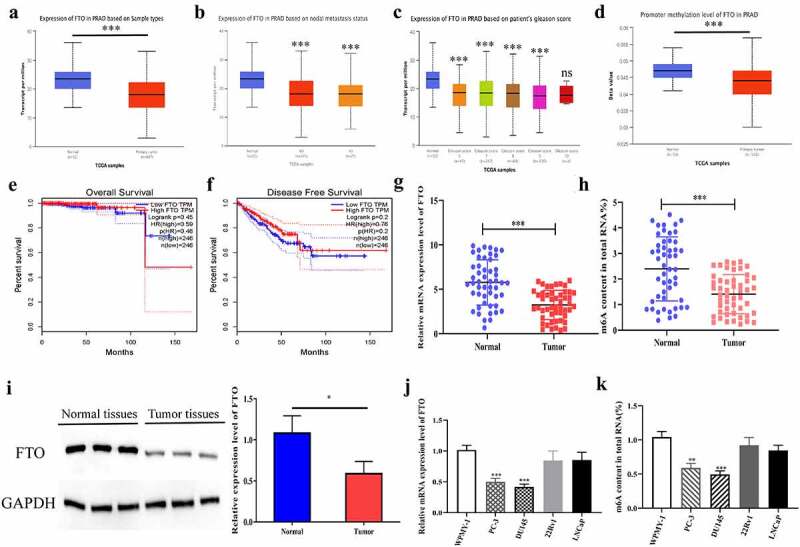
FTO was lowly expressed in PCa. (a). Assessment of FTO expression level in PCa tissues based on the Ualcan (http://ualcan.path.uab.edu/analysis.html) database. (b). The expression level of FTO in prostate cancer based on nodal metastasis status was obtained from the UALCAN database. (c). The expression level of FTO in prostate cancer based on patient Gleason score was obtained from the UALCAN database. (d).Assessment of promoter methylation level of FTO in PCa tissues based on the Ualcan database. (e). The associations between FTO expression and OS of PCa patients by analyzing TCGA. (f). The associations between FTO expression and DFS of PCa patients by analyzing TCGA. (g). Determination of FTO expression level in 50 pairs of PCa tissues via qRT-PCR. (h). Detection of m6A level of FTO in 50 pairs of PCa tissues via methylation. (i). Determination of FTO protein level in PCa tissues via Western blot. (j). Determination of FTO expression level in PCa cells via qRT-PCR. (k). Detection of m6A level of FTO in PCa cells via methylation. **P < 0.01; ***P < 0.001; ns no significant difference.

To explored the potential role of FTO in PCa, we predicted the expression level of FTO in PCa on bioinformatics websites, which yielded that the expression level of FTO in 497 pairs of tumor tissues was significantly lower than that in 52 pairs of normal tissues (Figure 1a). To investigate the relationship between FTO expression and clinicopathological parameters in PCa, we analyzed the UALCAN database and found that lower FTO expression was correlated with lymph node metastasis and a high Gleason score (Figure 1b, c). Subsequently, we predicted the promoter methylation level of FTO in PCa tissues on bioinformatics websites, and uncovered that PCa tissues had a dramatically lower methylation level of FTO than normal tissues (Figure 1d). Besides, we also discovered that the expression level of FTO had no correlation with total survival (OS) time and disease-free survival (DFS) time in PCa patients (Figure 1e, f). Further, we verified the expression level of FTO in 50 pairs of PCa tissues by qRT-PCR. As revealed in Figure 1g, the expression level of FTO in tumor tissues was dramatically lower than that in normal tissues. Moreover, we also found that PCa tissues had a lower m6A level of FTO than that in normal tissues (Figure 1h).In the meantime, the protein expression level of FTO in PCa was examined by Western blot assay. As shown in Figure 1i, it appeared that the protein expression level of FTO in PCa tissues was also lower than that in normal tissues. Subsequently, we detected the mRNA and m6A expression levels of FTO in normal prostate cells and PCa cell lines. The results uncovered that the mRNA and m6A levels of FTO in PCa cell lines were lower than those in normal cells, and PC-3 and DU145 with dramatically differential expression levels were selected for follow-up experiments (Figure 1j, k).

### FTO expression level had a relevance to the prognosis of PCa patients

**Figure 2.**
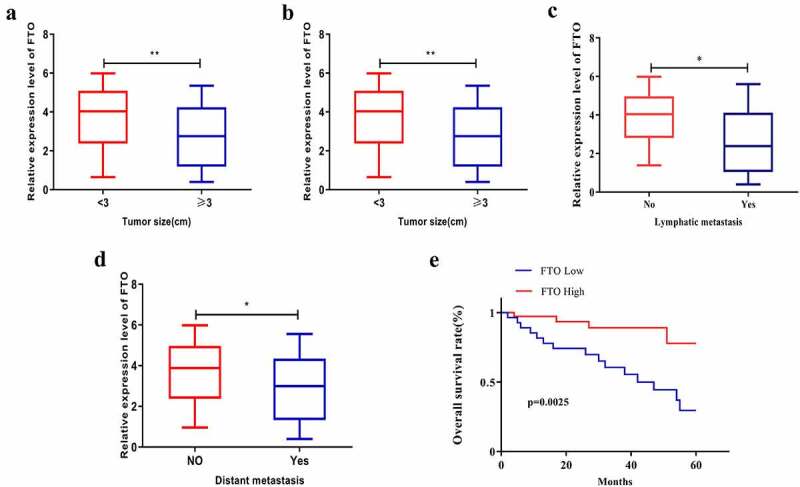
FTO expression level interrelated the clinical characteristics of PCa patients. (a). Assessment of expression level of FTO in different tumor size of PCa patients. (b). Analyze the expression level of FTO in different TNM stages of PCa patients. (c). Assessment of the interrelation between FTO expression level and lymph node metastasis of PCa patients. (d). Assessment of the interrelation between FTO expression level and distant metastasis of PCa patients. (e). Assessment of the interrelation between FTO expression level and overall survival rate of PCa patients. *P < 0.05; **P < 0.01.

Bioinformatics analysis was conducted to explore the relationship between FTO expression level and clinical features of PCa, which showed that FTO expression level was relevant to the Gleason score of PCa patients. As shown in Figure 2a, PCa patients with tumor diameter < 3 cm expressed FTO at a notably higher level than those with a tumor diameter ≥ of 3 cm (Figure 2b). Additionally, the FTO level in PCa patients at TNM stage I+ II was higher than that in patients at TNM stage III+IV (Figure 2c), and patients with low FTO expression had a high tendency to suffer lymph node metastasis, which was further confirmed by bioinformatics websites (Figure 2d). Meanwhile, distant metastasis of PCa appeared to be more likely to occur in patients with low FTO expression than in those with high FTO expression (Figure 2e). Subsequent assessment of the impact of FTO on the overall survival rate of PCa demonstrated that grossly more patients with high FTO expression survived than those with low FTO expression (Figure 2e).

### Downregulation of FTO contributed to the proliferation, migration, invasion and tumor growth of PCa cells

**Figure 3.**
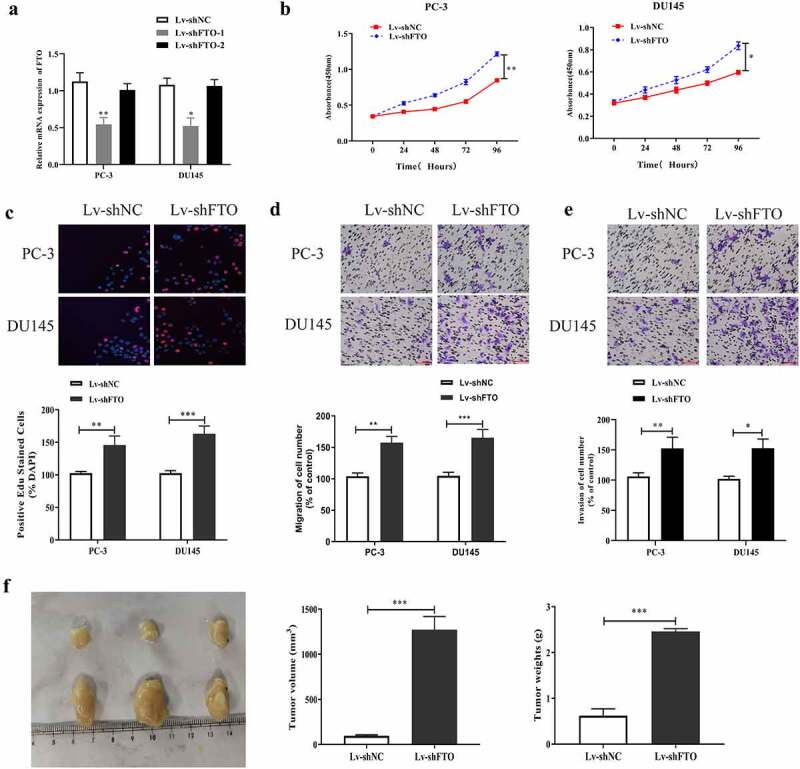
Decrement of FTO promoted PCa cells to proliferate, migrate and invade and tumor growth. (a). Examination of FTO expression level in PCa cells undergoing Lv-shFTO transfection via qRT-PCR. (b). Determination of absorbance at 450 nm of PCa cells undergoing Lv-shFTO transfection via CCK8 experiment. (c). Examination of EdU positive rate of PCa cells undergoing Lv-shFTO transfection via EdU experiment (200×). (d). Examination of impact of FTO decrement on the ability of PCa cells to migrate via transwell migration experiment (200×). (e). Examination of impact of FTO decrement on the ability of PCa cells to invade via transwell invasion experiment (200×). (f). The tumor growth rate of nude mice injected with Lv-shFTO transfected PCa cells was detected by tumor carrying test. *P < 0.05; **P < 0.01; ***P < 0.001.

First, we examined the impact of FTO on PCa malignant phenotypes by downregulating FTO expression in PCa using lentivirus packaging shRNAs (LV-shRNAs), and detected the interference efficiency by qRT-PCR. The results (Figure 3a) showed that FTO expression levels in PC-3 and DU145 cells transfected with LV-shFTO-1 were lower than those in cells transfected with shNC. In contrast, no prominent changes were observed in the expression of FTO in PC3 and DU145 cells transfected with LV-shFTO-2; therefore, we used LV-shFTO-1 with high transfection efficiency in cell experiments. Second, we utilized the CCK-8 assay to measure the absorbance values of PC3 and DU145 cells treated with LV-shFTO at 450 nm. It was observed that the absorbance values at 450 nm of the two cell lines transfected with LV-shFTO markedly increased compared to the absorbance of those transfected with shNC (Figure 3b). We further carried out the EdU assay to test the proliferation abilities of the two cell lines undergoing LV-shFTO transfection, and noted that they exhibited a dramatic EdU-positive rate compared to those undergoing shNC transfection (Figure 3c). Next, we tested the migration and invasion abilities of PC3 and DU145 cells transfected with LV-shFTO using a Transwell assay. As shown in Figure 3d, E, PC3 and DU145 cells transfected with LV-shFTO had stronger migration and invasion abilities than those transfected with shNC. After that, we performed *in vivo* animal experiments to measure the tumor diameter in nude mice after 3 weeks of growth, and the results showed that the tumor diameter of nude mice transfected with LV-shFTO was markedly larger than that of nude mice transfected with shNC (Figure 3f). These findings imply that the downregulation of FTO is beneficial for the malignant phenotypes of PCa.

### Overexpression of FTO suppressed the proliferation, migration, invasion and tumor growth of PCa cells

**Figure 4.**
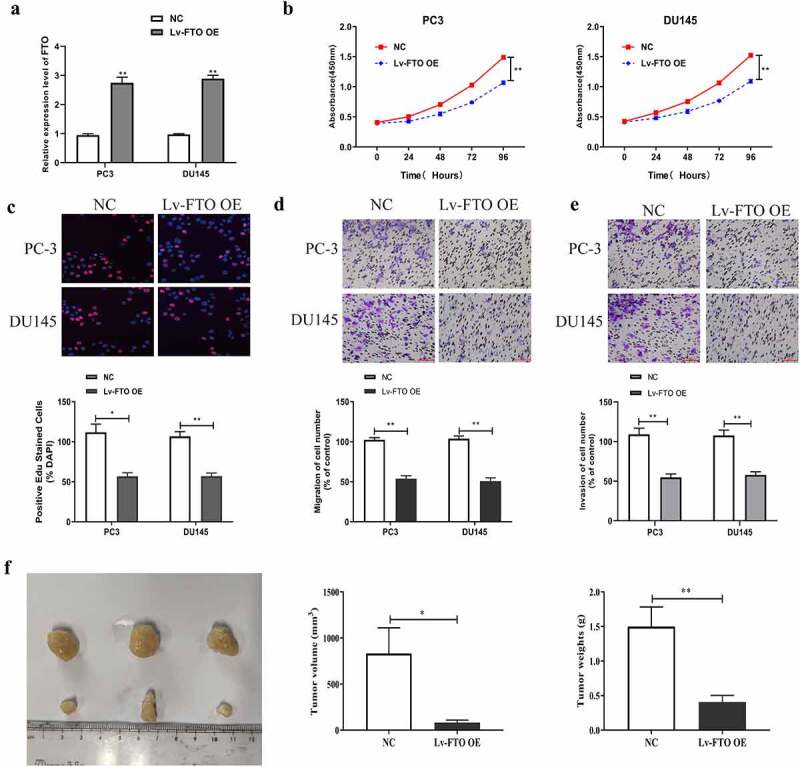
FTO overexpression suppressed PCa cells to proliferate, migrate and invade and tumor growth. (a). Examination of FTO expression level in PCa cells undergoing Lv-FTO OE transfection via qRT-PCR. (b). Determination of absorbance at 450 nm of PCa cells undergoing Lv-FTO OE transfection via CCK8 experiment. (c). Determination of EdU positive rate of PCa cells undergoing Lv-FTO OE transfection via EdU experiment (200×). (d). Examination of impact of FTO up-regulation on the ability of PCa cells to migrate via transwell migration experiment (200×). (e). Examination of impact of FTO up-regulation on the ability of PCa cells to invade via transwell invasion experiment (200×). (f). The tumor growth rate of nude mice injected with Lv-FTO OE transfected PCa cells was detected by tumor carrying test. *P < 0.05; **P < 0.01.

To further investigate the influence of FTO expression level on PCa malignant phenotypes, we increased FTO expression in PCa using lentiviral overexpression plasmid (LV-FTO OE), and detected transfection efficiency by qRT-PCR. As shown in Figure 4a, FTO expression levels in PC-3 and DU145 cells undergoing LV-FTO OE transfection were higher than those in the cells undergoing NC transfection. Second, we carried out a CCK-8 assay to measure absorbance values at 450 nm of PC3 and DU145 cells receiving LV-FTO OE transfection. Absorbance values at 450 nm of the cells after LV-FTO OE transfection dramatically dropped down relative to those of cells after NC transfection (Figure 4b). Third, we also tested the proliferation abilities of PC3 and DU145 cells transfected with LV-FTO OE by EdU experiment, and found that the EdU-positive rates of those cells were lower than those of cells transfected with NC (Figure 4c). We then tested the migration and invasion abilities of PC3 and DU145 cells transfected with LV-FTO OE using the Transwell assay. The results (Figure 4d, e) showed that the two cell lines transfected with LV-FTO OE had markedly weaker migration and invasion abilities than those transfected with NC. After that, we measured the tumor diameter in nude mice that had grown for 3 weeks, and the results showed that the tumor diameter of nude mice injected with PCa cells transfected with LV-FTO OE was significantly smaller than that of nude mice transfected with NC (Figure 4f).

### FTO could control m6A level of MC4R

**Figure 5..**
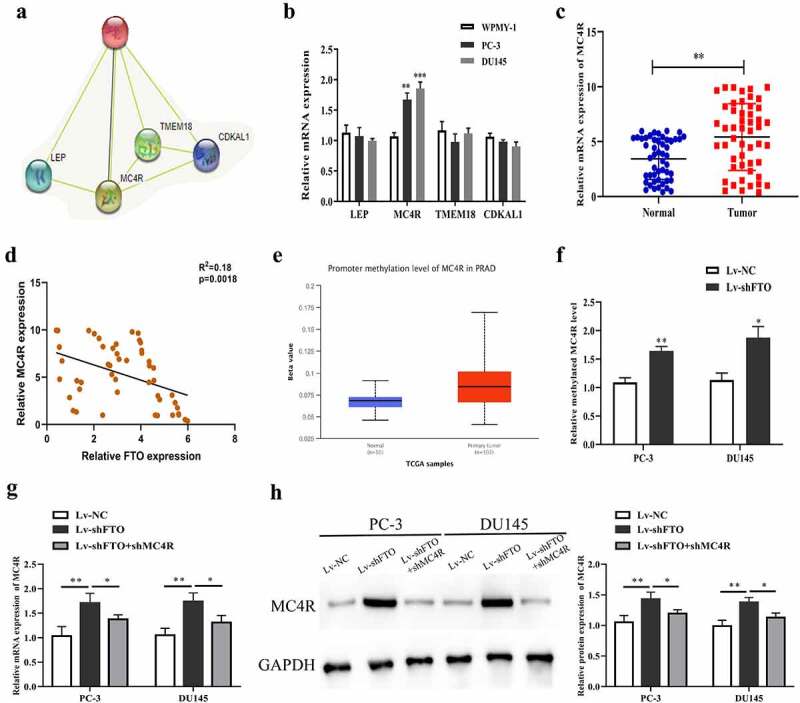
FTO exhibited negative interrelation with MC4R expression level. (a). Prediction of RNA binding proteins that may interact with FTO by String (https://string-db.org/) database. (b). The expression of RNA binding protein in PCa cells was detected by qRT-PCR. (c). Determination of MC4R expression level in 50 pairs of PCa tissues via qRT-PCR. (d). Assessment of the interrelation between FTO and MC4R expression level using Pearson method. (e). Prediction of the methylation modification of MC4R via Ualcan prediction website. (f). M6A level of MC4R in PCa cells undergoing Lv-shFTO transfection via methylation. (g). Examination of MC4R expression level in PCa cells undergoing Lv-shFTO and shMC4R transfection via qRT-PCR. (h). Examination of MC4R protein expression level in PCa cells undergoing Lv-shFTO and shMC4R transfection via Western blot. *P < 0.05; **P < 0.01.

We predicted 4 RNA-binding proteins (RBPs) (LEP, MC4R, TMEM18 and CDKAL1) that may interact with FTO through bioinformatics websites to study the molecular mechanism of FTO in PCa (Figure 5a). After that, we performed qRT-PCR to determine the mRNA expression level of the 4 RBPs. As revealed in Figure 5b, the mRNA expression level of MC4R in PC-3 and DU145 cells rose up prominently, and this level was then detected in 50 pairs of PCa tissues, which yielded that it was higher in PCa tissues than in normal tissues (Figure 5c). And we employed Pearson method to assess the correlation between MC4R and FTO expression level, which yielded that the two had a remarkable inverse correlation (Figure 5d). In the meantime, it was uncovered through bioinformatics websites that the methylation level of MC4R in PCa also rose up dramatically (Figure 5e). Subsequent analysis demonstrated that MC4R methylation level after decreasing FTO was markedly raised (Figure 5f). Further, qRT-PCR and Western blot assays uncovered that the mRNA and protein expression levels of MC4R in PC-3 and DU145 cells undergoing shFTO transfection notably promoted relative to those in the cells transfected with shNC, while declined when con-transfected with shMC4R (Figure 5g, h). To explore the mechanism of MC4R in PCa, we used m6avar (http://m6avar.renlab.org/index.html) to analyze and found that there are multiple m6A modification sites in the MC4R sequence, mainly located in the 3ʹUTR of MC4R (Figure S1A). Through MeRIP-qPCR detection, we found that overexpression of FTO in PC-3 and DU145 cells could inhibit MC4R mRNA (Figure S1B).To determine the effect of m6A modification on the expression of MC4R, we constructed wild-type or mutant MC4R. For the mutant form of MC4R, the adenosine bases in the m6A consensus sequence (RRACH) were replaced by cytosine, so as to cancel the m6A modification (Figure S1C). Through the double luciferase reporter gene assay, it was found that the relative luciferase activity of MC4R 3ʹ-UTR with wild-type m6A site was significantly increased after FTO overexpression, while inhibition of FTO could inhibit the luciferase activity of MC4R 3ʹ-UTR. However, the relative luciferase activity of MC4R 3ʹ-UTR with mutant m6A site has nothing to do with the expression of FTO (Figure S1D, E).

### High expression of FTO partially limited the promotion of high expression of MC4R on PCa malignant phenotypes

**Figure 6.**
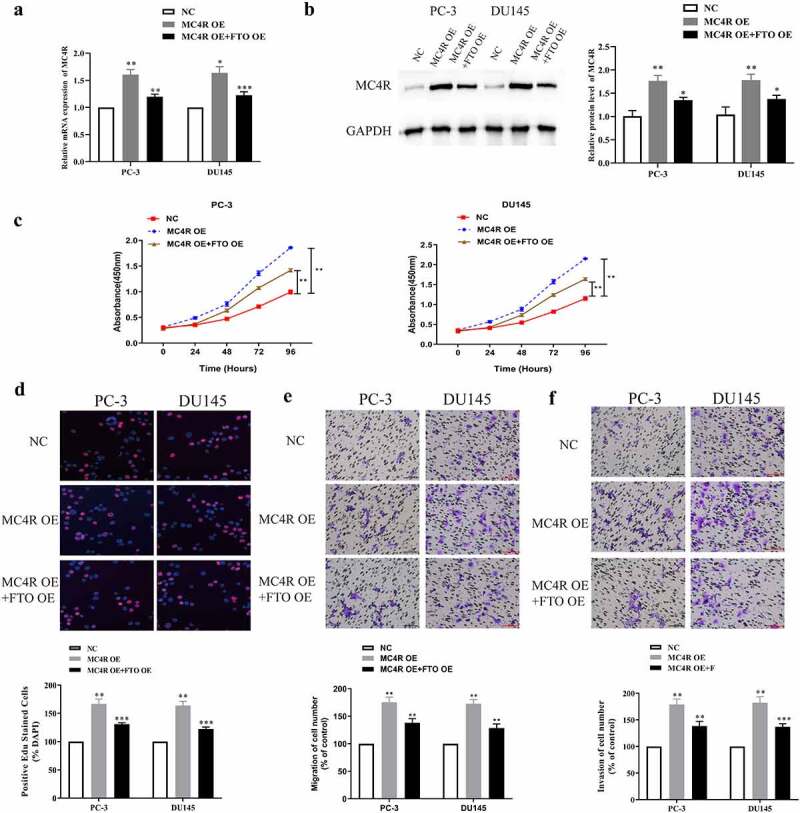
High-expression FTO partway reversed the malignant phenotype of PCa mediated by high-expression MC4R. (a). Examination of MC4R expression level in PCa cells undergoing MC4R OE and FTO OE transfection via qRT-PCR. (b). Examination of MC4R protein expression level in PCa cells undergoing MC4R OE and FTO OE transfection via Western blot. (c). Determination of absorbance at 450 nm of PCa cells undergoing MC4R OE and FTO OE transfection via CCK8 experiment. (d). Examination of detected the EdU positive rate of PCa cells undergoing MC4R OE and FTO OE transfection via EdU experiment (200×). (e). Examination of the ability of PCA cells undergoing MC4R OE and FTO OE transfection to migrate via transwell migration experiment (200×). (f). Examination of the ability of PCa cells undergoing MC4R OE and FTO OE transfection to invade via Transwell invasion experiment (200×). *P < 0.05; **P < 0.01.

To further probe the joint action of FTO and MC4R on PCa, we co-transfected MC4R OE and FTO OE into PC-3 and DU145 cells and tested the transfection efficiency via qRT-PCR. The mRNA expression level of *MC4R* after co-transfection with MC4R OE and FTO OE was lower than that after transfection with MC4R OE alone, but was still higher than that after NC transfection (Figure 6a). According to the Western blot assay, MC4R expressed more proteins in PCa cells co-transfected with MC4R OE and FTO OE than in those transfected with NC, but expressed less than in those transfected with MC4R OE (Figure 6b). Next, we used the CCK-8 assay to measure the absorbance values at 450 nm of PC-3 and DU145 cells co-transfected with MC4R OE and FTO OE, and found that overexpressed FTO could partially limit the high absorbance values of cells highly expressing MC4R at 450 nm (Figure 6c). Besides, it was revealed that FTO overexpression partially limited the promotion of MC4R high expression on the high EdU-positive rate of PC-3 and DU145 cells (Figure 6d). Additionally, we used the Transwell assay to test the migration abilities of PC-3 and DU145 cells after co-transfection with MC4R OE and FTO OE. The high expression of FTO partially limited the promotion of MC4R on the migration and invasion abilities of PC-3 and DU145 cells (Figure 6e, f).

## Discussion

PCa is the second most common cancer in men worldwide and is also the most common cancer in men in developed countries [29]. The occurrence of PCa is related to age, race, geographical location, genetic history, family history, androgens, metabolic syndromes, living habits, diets, genes, etc [30]. At the early stage, PCa is treated by radical surgery, achieving favorable efficacy; however, owing to the lack of obvious symptoms, PCa is not diagnosed until it is already at an advanced stage in most patients with poor prognosis [31]. Hence, it is necessary to identify more influential molecular targets that are beneficial for the diagnosis and treatment of PCa.

M6A was first proposed in 1974, and it was later found that m6A methylation widely exists in different tissues and organs [32]. It is dynamically regulated by various regulatory factors and strongly supports mRNA splicing, translation, and stability, as well as various normal biological processes, such as tissue development, stem cell self-renewal and differentiation, heat shock, and DNA damage response [33]. FTO, also known as AlkBH9, belongs to the non-heme KGFe(II)/α-KG-dependent dioxygenase ALKB family (ABH1-9), is located on chromosome 16q12.2 [34], and is regarded as the first RNA demethylase [16]. The discovery of FTO reinforces the finding that m6A methylation is a reversible dynamic process, which not only mediates the stability, splicing, transport, localization, and translation of mRNA, but also regulates the processing of miRNA and the interaction of RBPs [35]. FTO is capable of oxidizing demethylated m-3 T and m-3 U in single-stranded DNA and single-stranded RNA in vitro [36]. In recent years, an increasing number of studies have been conducted on FTO in human malignancies. For example, Zhao et al. revealed that FTO is beneficial for the growth of ovarian cancer cells by stimulating cell proliferation, inhibiting apoptosis, and activating autophagy [37]. Zhang et al. showed that FTO supports endometrial cancer metastasis by modifying the m6A of *HOXB13* mRNA and activating the Wnt signaling pathway. Tian et al [38]. discovered the ability of FTO to mediate the metastasis of thyroid cancer and its correlation with the prognosis of thyroid cancer patients [39].

In the current study, we found that the expression level of FTO was low in PCa tissues by bioinformatic analysis. It was speculated that FTO might function as a tumor suppressor gene in PCa, which was confirmed by qRT-PCR and Western blot assays. Additionally, the analysis of the relationship between FTO expression level and clinical characteristics of PCa patients revealed that patients with low expression levels of FTO had a significant correlation with tumor metastasis and gleason score, which indicated that low expression FTO might be related to poor prognosis in PCa patients. However, the expression level of FTO did not show association with PCa patients OS rate and DFS rate. In future studies, we will follow up the patients included in the study and analyze the correlation between FTO expression and survival prognosis. Moreover, we downregulated the expression level of FTO in PCa using small interfering RNA, and CCK-8, EdU, and Transwell assays revealed that the proliferation, migration, and invasion abilities of PCa cells after shFTO transfection were stronger than those after shNC transfection. FTO downregulation was beneficial for PCa growth, but the opposite result was observed after FTO overexpression. Hence, we speculated that FTO might act as a tumor suppressor gene in PCa and inhibited the malignant phenotypes of PCa.

It has been previously shown that MC4R play certain biological functions in obesity [40], and they are also associated with breast cancer [41], colon cancer [42] and endometrial cancer [43]. However, the potential role and mechanism of MC4R in PCa remains unknown. In this study, bioinformatics websites revealed a co-expression relationship between FTO and MC4R, and a slew of correlation experiments revealed the capacity of FTO to control m6A levels of MC4R. Additionally, FTO and MC4R expression levels exhibited a grossly negative relationship, and the torsion test further verified that high FTO expression partially reversed the promotion of high MC4R expression on PCa malignant phenotypes. Moreover, we also found that MC4R exerted m6A modification sites and FTO could lead to its demethylation.

Due to time and cost constraints, this study still has many shortcomings. First of all, we also need to collect more PCa specimens to further verify the correlation between FTO and clinical characteristics, as well as the correlation between FTO and the survival prognosis of PCa patients. Secondly, we should detect the possible downstream targets of FTO through MeRIP-sequence technology, rather than through the method of bioinformatics prediction. Moreover, the expression of mRNA is regulated by downstream recognition proteins. This study did not explore which downstream recognition proteins can recognize the methylation sites of MC4R and further regulate its expression.

## Conclusion

*FTO* acts as a tumor suppressor gene in PCa, and its expression level is relevant to the prognosis of patients with PCa. Additionally, it can interact with MC4R through m6A modification and control the proliferation, migration, and invasion abilities of PCa cells.
